# Influence of Interface Inclination Angle and Connection Method on the Failure Mechanisms of CFRP Joints

**DOI:** 10.3390/polym18030344

**Published:** 2026-01-28

**Authors:** Junhan Li, Afang Jin, Wenya Ruan, Junpeng Yang, Fengrong Li, Xiong Shu

**Affiliations:** 1 College of Mechanical Engineering, Xinjiang University, Ürümqi 830047, China; 107552301375@stu.xju.edu.cn (J.L.); 107552404354@stu.xju.edu.cn (W.R.); 107552304357@stu.xju.edu.cn (J.Y.); 107552304272@stu.xju.edu.cn (F.L.); 2 Department of Mechanical Engineering, Hunan Institute of Engineering, Xiangtan 411104, China; shux@hnie.edu.cn

**Keywords:** composite material, connection interface, hybrid connection, bonding, damage mechanism

## Abstract

Carbon fiber reinforced polymers (CFRPs) are widely used in aerospace and wind power applications, but the complex failure mechanisms of their connection structures pose challenges for connection design. This study aims to investigate the influence of bonding interface inclination angle and connection method on the failure mechanisms of CFRP joints under bending loads. The study investigated two design parameters: the joint geometry of the bonding interface (single-slope, transition-slope, and single-step) and the connection methods (bonding, bolting, and hybrid bonding–bolting). Finite element simulations analyzed the mechanical performance and failure modes under different design parameters. Bending tests validated the mechanical properties of the joint interface, validating the effectiveness of the numerical simulation. The study found that under bonded connections, the bending load increased with the slope of the connection interface, with improvements of 21.87% and 39.75%, respectively. The main reason is stress concentration caused by sharp geometric discontinuities. The hybrid connection had the highest peak load, with improvements of 38.38% and 43.91% compared to the other connection methods. Hybrid bonding–bolting connections further optimized structural performance and damage tolerance. This study reveals the damage mechanisms of the bonding interface and provides a reliable prediction method for aerospace and wind turbine blade applications.

## 1. Introduction

Carbon fiber reinforced polymers (CFRPs) are widely used in various fields due to their excellent overall performance. Compared to traditional metals such as aluminum alloys, titanium, and steel, CFRP materials offer higher stiffness and strength with lower weight [1]. In addition, CFRP materials have excellent corrosion resistance and are widely used in various industries, including aerospace, automotive, marine, and wind turbine blades [2,3]. Despite their many advantages, any design and optimization activities for lightweight and safe structures using CFRP materials should aim for the global optimal solution whenever possible. The main joining techniques that can be used for composite parts, depending on the conditions, are bonding [4,5], bolting [6], and bonding/bolting [7,8].

In bonded structures, loads in one bonding layer are transferred through the bonding layer to another bonding layer. The efficiency of load transfer depends on the joint design, adhesive properties, and adhesive–substrate interface [9,10]. Glued joints have their limitations, especially their sensitivity to environmental factors (e.g., temperature, humidity) [11], which may lead to debonding of the adhesive layer and degradation of structural properties in long-term service [12]. In bonded joints, the complex stress distribution and high stress concentrations generated at the free edges may lead to debonding failures [13,14]. In addition, the lack of redundant protection in glued joints may lead to sudden damage of the joint in case of failure of the adhesive layer [15,16]. Previous studies have either focused mainly on the effect of the adhesive layer on the strength of the joint structure or only considered the damage of composite panels at the lap interface [17,18,19], without combining the two for an in-depth study. With the continuous in-depth research on composite material bonding technologies, more attention has been focused on how to optimize the adhesive interface design to improve the overall strength and safety of the structure [19,20,21,22]. Bolted connections in composite materials are prone to local stress concentrations, which can reduce the load-bearing capacity of the structure. On the other hand, using only fasteners (bolts, rivets, and pins) to connect composite structures is not ideal [23]. Furthermore, the efficiency of bolted connections in brittle composites is lower than that of metal bolted connections [24]. Although the most common damage mode for bolted connections is load-bearing damage, the strongest damage mode per unit of laminate width is always net section tension [25]. Bolted connections are usually avoided.

To address the limitations of both adhesive and bolted connections, hybrid adhesive-bolted joints have gradually become an important connection method. Generally speaking, compared to purely bonded or mechanically fastened joints [26], hybrid connections exhibit better static and fatigue performance. Research by Chowdhury et al. [27] indicates that hybrid adhesive-bolted joints show longer service life in fatigue tests and have higher durability and damage tolerance compared to purely adhesive or bolted connections. Blier et al. [28] studied the tensile behavior of hybrid adhesive-bolted connections through experiments and finite element analysis. The research found that hybrid adhesive-bolted joints exhibit lower stress concentration and better geometric retention compared to purely bolted connections. Qin et al. [29] explored the fatigue damage behavior of CFRP-embedded bolt assemblies under three-point bending. It was shown that composite joints with pre-embedded bolts exhibited significant interfacial damage under low-cycle loading, and their fatigue life was closely related to the bolt diameter and clamping force. Yousefi Kanani et al. [30] reviewed the failure mechanisms of hybrid adhesive-bolted joints under different loading conditions, proposed geometric and material improvements to optimize the performance of dissimilar joints, and discussed the impact of different designs on joint performance. Although the impact of bonding interface design on stress distribution and failure has been studied, and the advantages of hybrid connections over single connection methods are recognized, few studies have explored how these two factors interact under bending loads. [31]. This study aims to fill this gap by investigating the combined effect of bonding interface inclination angle and hybrid connection methods on the mechanical performance and failure modes of CFRP joints, providing new insights into the design and optimization of CFRP structures.

This paper presents an experimental study on the bending load of joint geometry under two different design parameters, and proposes a comprehensive finite element model that combines the bond layer, the CFRP damage process, and the change in the bolt force, and adopts the numerical prediction method of a two-step analysis process, which reveals in detail the damage mechanism of the bonding interface and the evolution process of the damage. The mechanical properties of the bonding interface and the relationship between load and displacement are also investigated, and the experimental measurements are compared with the numerical results, which are in good agreement and prove the validity of the established model. Finally, the fracture morphology and internal damage evolution are presented and discussed.

## 2. Experimental Setup and Procedure

### 2.1. Materials and Properties

The experimental material used in this study is T700 carbon fiber/epoxy resin composite laminate produced by Jiangsu Boshi Carbon Fiber Technology Co., Ltd. (Nanjing, China), which adopts a non-woven design, and the curing and molding of prepreg is performed in a hot press tank under high temperature and high-pressure conditions. The density is 1.80 g/cm^3^, 48 layers comprise the symmetrical layup, and the specimen layup sequence is [45/0/90/−45]_6s_, and the single layer thickness is 0.15 mm. The mechanical properties are shown in Table 1 Where subscripts 11, 22, and 33 indicate longitudinal, transverse, and normal directions, subscripts 12 and 13 denote the two in-plane shear directions, and subscript 23 denotes the out-of-plane shear direction. X, Y, and S are the longitudinal, transverse, and shear strengths, respectively, and subscripts T and C denote the tensile and compressive strength, respectively. E and G are Young’s modulus and the shear modulus, and V is Poisson’s ratio. $G_{fc}^{C}$, $G_{mt}^{C}$ and $G_{mc}^{C}$ are the damage fracture energies of the fibers and the substrate, respectively. $K_{ii}$(i=n, s or t) is the stiffness of the separation of the traction stress from the three directions. The subscript n denotes the normal direction, and the subscripts s and t denote the in-plane and out-of-plane shear directions.

**Table 1:** Mechanical properties of composite materials.

Mechanical Properties		Average Values
Young’s modulus	$E_{11}$ (GPa)	140
	$E_{22}$ $(\mathrm{GPa}),\;E_{12}$ (GPa)	8.40
Shear modulus	$G_{12},G_{13}$ (GPa)	4.50
	$G_{23}$ (GPa)	3.50
Poisson’s ratio	$\nu_{12},\;\nu_{13}$	0.30
	$\nu_{23}$	0.35
Longitudinal tensile strength	$X_{T}$ (MPa)	2200
Longitudinal compressive strength	$X_{C}$ (MPa)	1250
Transverse tensile strength	$Y_{T}$ (MPa)	42
Transverse compressive strength	$Y_{C}$ (MPa)	175
Longitudinal shear strength	$\sigma_{n}$ (MPa)	121
Transverse shear strength	$\tau_{n}$ (MPa)	88
Mode I fracture energy	$G_{IC}$ (N/mm)	0.52
Mode II fracture energy	$G_{IIC}$ (N/mm)	0.92
Density	β (g/cm^3^)	1.76
Interface stiffness	$K_{nn},K_{ss},K_{tt}$ (N/mm^3^)	$2\times 10^{5}$

The adhesive used in this experiment is Araldite^®^ 2015 (Huntsman Advanced Materials (Switzerland) GmbH, Basel, Switzerland), which has high shear modulus, high toughness, low shrinkage, excellent corrosion resistance, and wide utility. The properties are shown in Table 2.

**Table 2:** Mechanical properties of adhesive Araldite^®^ 2015 [32,33].

Mechanical Properties	Value
Young’s modulus E /GPa	1.85
Shear modulus G /GPa	0.56
Poisson’s ratio ν	0.33
$\mathrm{Peak}\;\mathrm{normal}\;\mathrm{traction}\;\;T_{n,max}$/MPa	21.63
$\mathrm{Peak}\;\mathrm{shear}\;\mathrm{traction}\;T_{s,max},T_{t,max}\;$/MPa	17.90
$\mathrm{Mode}\;I\;\mathrm{fracture}\;\mathrm{energy}\;\;G_{IC}$/N/mm	0.43
$\mathrm{Mode}\;\mathrm{II}\;\mathrm{fracture}\;\mathrm{energy}\;G_{IIC}\;$/N/mm	4.70

### 2.2. Design of CFRP Laminate Joint Structures

The aim of this study is to investigate the influence of the bonding region of CFRP (carbon fiber reinforced composite) laminate lap structures on the three-point bending performance under different skew designs, and also to evaluate the trends of different connection methods on their mechanical responses. In order to ensure the consistency of the test and the comparability of the data, the specimen dimensions were designed according to ASTM D7337 [34] and ASTM D7264 [35] standards, and appropriately adjusted in accordance with the experimental requirements. The total length of the specimen is kept constant to minimize the influence of geometric scale variation on the test results.

**Figure 1:**
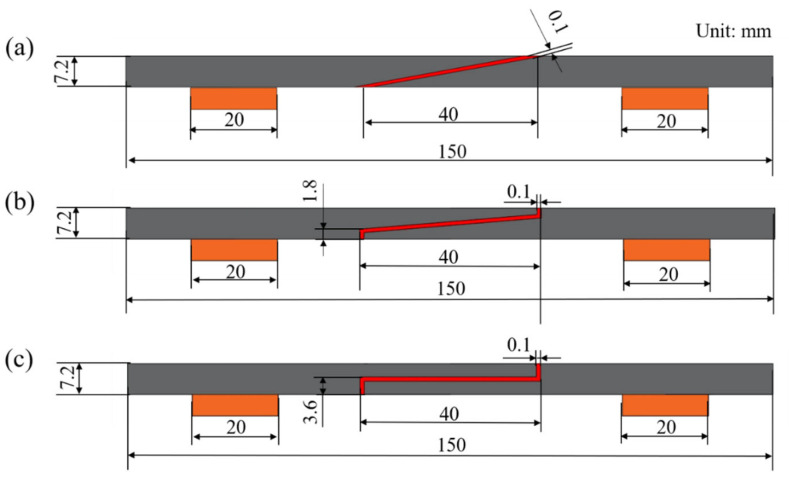
Schematic diagrams of cemented structures with different slopes (stepped): (**a**) single-slope, (**b**) transition-slope, (**c**) single-step.

The basic dimensions of all specimens were uniformly set as follows: length 150 mm, width 25 mm, and thickness 7.2 mm. To study the effect of slope on structural performance, three different adhesive structures with varying connection angles were designed, as shown in Figure 1. The length of the cut area was fixed at 40 mm, and the width was 25 mm. The three structures are defined as follows: single-slope structure, transition-slope structure, and single-step structure. Within the entire 40 mm cutting zone of the single-slope structure, the thickness gradually transitions from 7.2 mm to 0, with a corresponding bonding interface inclination angle of 10.2° (relative to the horizontal plane along the laminate’s length). In the transition-slope structure, the bonding interface consists of a 1.8 mm-high step section and a short ramp section with a 5.14° slope. The single-step type structure forms a single step at the joint bonding interface. Each laminate bonding member is thinned to half its original thickness in the overlap zone, creating a 3.6 mm-high step structure. The bonding areas are as follows: single-slope structure (1016 mm^2^), transition-slope structure (1094 mm^2^), and single-step structure (1180 mm^2^). The maximum area difference is 7.59%. It is worth noting that, to ensure experimental accuracy and adhesive symmetry, all specimens were designed with strict geometric symmetry in the cutting area. This structural symmetry helps achieve dimensionally consistent CFRP composite laminate assemblies after the overlap connection, thereby eliminating the stress concentration effects caused by dimensional deviations.

**Figure 2:**
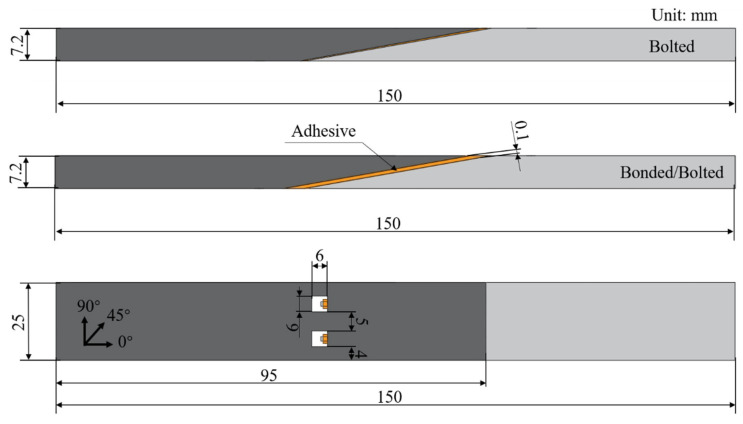
Bolted and hybrid connection configurations and dimensions.

In addition, based on the above results, the connection structure with the highest flexural strength was selected, and the failure modes, ultimate load carrying capacity, and energy dissipation characteristics of the three types of purely glued connections, bolted connections, and hybrid glued–bolted connections under three-point bending loads were studied and analyzed. The CFRP pre-bolted connection structure was designed with reference to the ASTM D6873 [36] standard, and 12 specimens were fabricated. in order to reveal the influence law of the connection method on the overall bending performance of the structure. Figure 2 illustrates the connection structure under the same lap angle. The bolted connection structure is fixed by two preloaded bolts with nominal bolt diameter D = 3 mm, hole diameter of 3 mm, and bolt spacing and side distance of 6 mm and 5 mm, respectively, to avoid end damage and stress concentration. Meanwhile, to improve the docking accuracy and repeatability, all bolt holes are processed by CNC machine tools. For a glue–bolt hybrid connection, structural adhesive is coated on the basis of the above bolt connection structure to form an adhesive–mechanical double bearing system. The overall dimensions of the specimens were standardized as 150 mm in length, 40 mm in length, and 25 mm in width of the overlap area to exclude the influence of dimensional differences on the bending response.

The pre-embedded bolts in the specimens were manufactured from 304 austenitic stainless steel and the matching nuts were 304 (A2-70) stainless steel. The tightening torque of the fasteners was 5.0 N·m (pre-tightened 2.0 N·m before curing and tightened again to 5.0 N·m after curing). The inner diameter of the bushing, d, was 3.126 to 3.202 mm, which was slightly larger than the bolt diameter, indicating that the clearance between the bolt and the bushing was small. The resulting diameter ratio d/D ≈ 1.042~1.067, and the pitch ratio S/D ≈ 1.33. The connection between the CFRP laminates was made by combining the embedded bushings and bolts. The mechanical properties are shown in Table 3.

**Figure 3:**
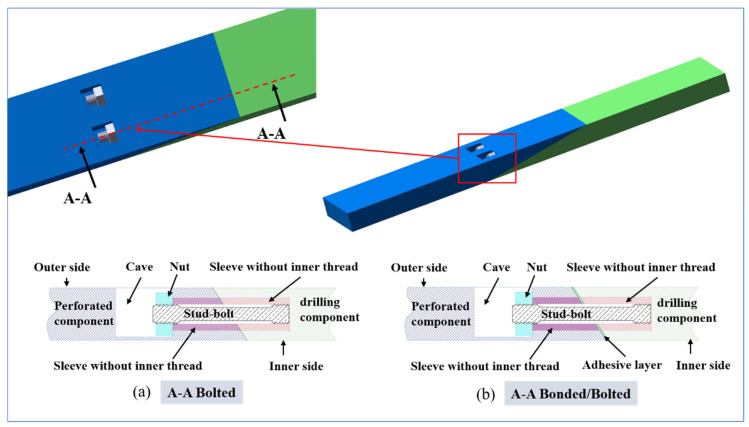
Global view of bolted and hybrid connections: (**a**) Bolted, (**b**) Bonded/Bolted.

The structural characteristics of the CFRP pre-embedded bolts are shown in Figure 3. As can be seen in the figure, the pre-bolted connection consists of two sections of composite plywood connected by bolts. Each bolted connection consists of a bolt, a hollow liner sleeve, and a nut. The ends of the bolts are machined with threads, and the inner surface of one liner sleeve is machined with threads at one end and unthreaded at the other.

### 2.3. Specimen Fabrication

**Figure 4:**
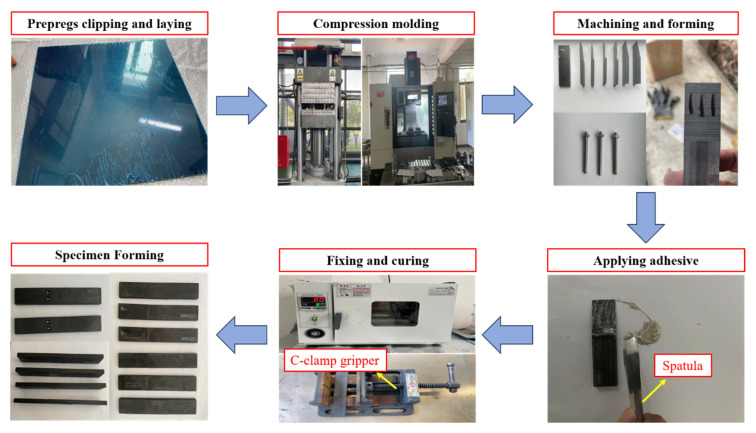
CFRP prototype fabrication process.

The experimental material was a T700 carbon fiber/epoxy composite laminate, and in order to meet the mold specifications, large-size prepregs with a thickness of 0.15 mm per layer needed to be cut into squares of 500 mm × 500 mm and subsequently stacked in the order of [45/0/90/−45]_6s_. A release agent is then applied to both sides of the finished preform to facilitate the subsequent demolding of the CFRP laminate. The prepregs were then placed in a hot press along with the molds, and the temperature was gradually increased to 120 °C over a period of 80 min, maintained at this temperature for 80 min, and subsequently allowed to cool naturally to room temperature prior to the demolding process. The obtained composite laminates were cut into specimens of 25 mm width and 150 mm length, making them suitable for the three-point bending test. After processing and molding, each specimen was quality assessed to ensure that there were no visible manufacturing defects. Before bonding two composite laminates, the bonding surface needs to be pretreated. During the bonding process, the adhesive is uniformly applied to the fracture to avoid the bonding layer being too thick or too thin, which will affect the mechanical properties and crack extension properties of the bonding. Additionally, a controlled thickness of adhesive layer was achieved by sprinkling a specified quantity of 0.1 mm diameter glass beads. In order to improve the fixation performance of the bonding interface, the composite fixation method of silica gel wrapping combined with tape winding is adopted. This design takes advantage of the thermal expansion properties of silicone material, which expands in volume under heating conditions, thereby increasing the constraint force on the bonding area and optimizing the stability of the overall structure. Subsequently, it is fixed with c-clamp and placed in the XMTA-L-5000 electric constant temperature drying oven. The bonding interface was cured by isothermal curing at 80 °C for 80 min to achieve a complete cross-linking reaction, thus ensuring that the bonding line was fully cured to meet the mechanical property requirements of the material system. After the curing process is completed, the specimen is placed in the electric constant temperature drying oven, and the gradient temperature reduction is realized by a 2 h slow cooling process, which effectively reduces the residual thermal stress due to the difference in the coefficient of thermal expansion. The structural adhesive residue in the overflow area of the cured specimen was removed by a sandpaper sanding process to avoid adverse effects on the subsequent tests. At least four test samples were prepared for each structure. Figure 4 depicts the process of making CFRP samples.

### 2.4. Quasi-Static Three-Point Bending Test

**Figure 5:**
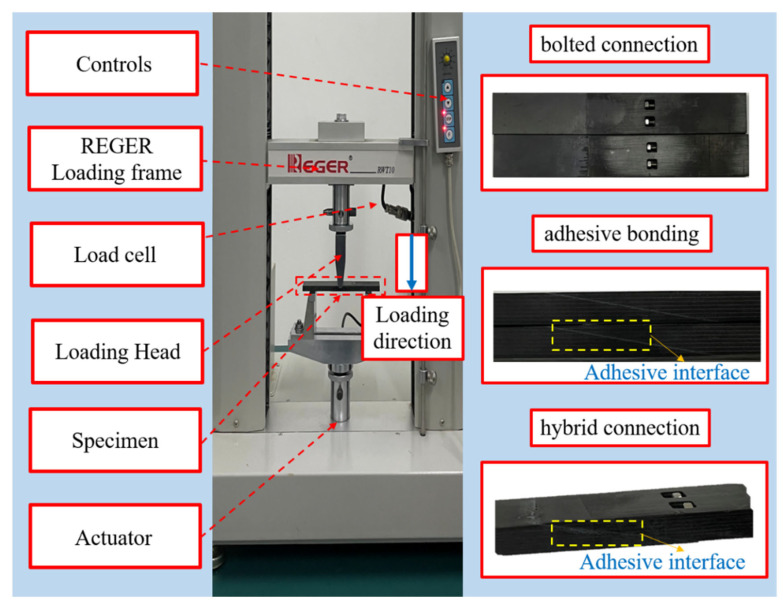
Three-point bending experimental diagram.

In this study, three-point bending experiments of CFRP composite laminates were carried out using a displacement control mode. Referring to the test standard ASTM D7264/D7264M-21 [35], a 50KN electronic universal testing machine was used to conduct three-point bending tests on all specimens. As shown in Figure 5, the testing machine includes components such as compression stage, transducer, fixture, and control system.

**Figure 6:**
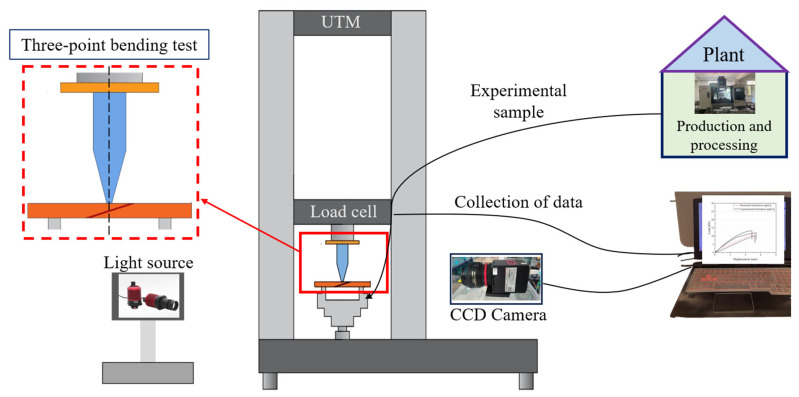
Roadmap of experimental steps.

The displacement control of the testing machine was carried out at a constant speed of 1.5 mm/min, and the three parameters of load–displacement–time were recorded synchronously through the built-in data acquisition system of the universal testing machine. The sampling frequency was set to 20 Hz to ensure data continuity. It is important to note that the equipment is equipped with a rigid support platform and a cylindrical press head, with a support span of 115 mm. The specimens were placed horizontally on the test bench, ensuring that the specimen’s centerline aligned with the loading centerline. To eliminate systematic errors caused by the contact gap during the initial loading stage, the contact interface was adjusted in advance through a preloading program, which removed the influence of the gap on the experiment. After these pre-treatment steps, the testing machine applied the load until the adhesive layer was destroyed. The load–displacement relationship at the failure of the adhesive layer was recorded during the test. Figure 6 illustrates the entire experimental procedure. All testing procedures were performed at room temperature with at least four specimens in each tensile to avoid accidental errors. This experimental configuration realizes the simultaneous acquisition of macroscopic mechanical response and structural evolution, which provides multi-dimensional experimental data support for exploring the bending damage mechanism in the bonding region of composite laminates.

## 3. Numerical Simulation Analysis

### 3.1. Damage Mechanism Research

**Figure 7:**
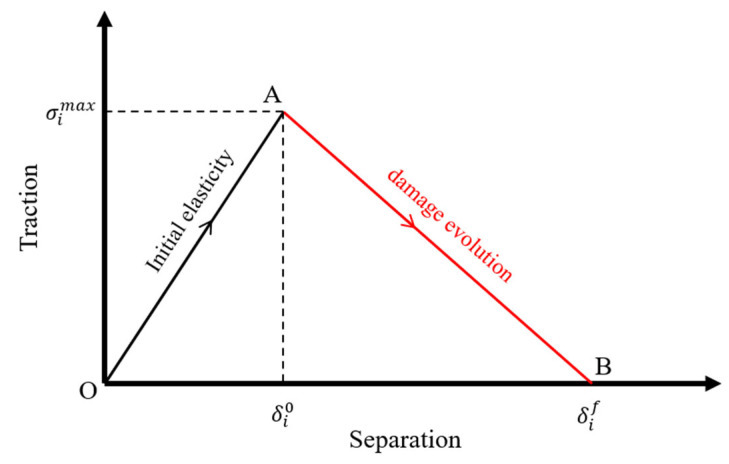
Bilinear cohesive zone model.

The cohesive zone model (CZM) with traction–separation jump curves of different shapes is considered the most effective method for simulating the damage process of the adhesive layer [37]. CZM combines strength-based failure criteria and fracture mechanics-based damage evolution criteria to predict damage, concentrating the degradation mechanism on a discrete line or plane in front of the actual crack tip [38]. In this study, a bilinear CZM in ABAQUS is employed to simulate the damage initiation and propagation of the adhesive interface at the connection of composite laminates under bending forces. The bilinear CZM consists of two stages: initial elasticity (OA section) and damage evolution (AB section) [39], as shown in Figure 7. In the initial elasticity stage, the stress increases linearly with the relative displacement of the cohesive element. When the stress reaches the maximum point $\sigma_{i}^{max}$ (i=n, s or t), i.e., when the relative displacement reaches $\sigma_{i}^{0}$ (i=n, s or t), the cohesive element satisfies the initial damage criterion, and damage initiation occurs within the bondline. In the damage evolution stage, as the relative displacement continues to increase, the stress decreases linearly until it reaches zero. The cohesive element fails completely when the relative displacement reaches $\sigma_{i}^{f}$ (i=n, s or t) where $\sigma_{i}^{f}$ are the displacement values in the normal and tangential directions, respectively. The subscripts n, s, and t represent the normal direction and the two tangential directions of the cohesive element.

When the CZM is in the elastic stage ($\delta_{i}$ < $\delta_{i}^{0}$, i=n, s or t), the constitutive relation is as follows [40]:

(1) $$\left\{\begin{array}{c} \sigma_{n}\\ \sigma_{s}\\ \sigma_{t} \end{array}\right\}=\left[\begin{array}{ccc} K_{nn} & K_{ns} & K_{nt}\\ K_{ns} & K_{ss} & K_{st}\\ K_{nt} & K_{st} & K_{tt} \end{array}\right]\left\{\begin{array}{c} \delta_{n}\\ \delta_{s}\\ \delta_{t} \end{array}\right\}$$

where $\sigma_{n},\;\sigma_{s},\;{\;\sigma }_{t}$ are the normal stress, shear stress, and tearing stress, respectively, and $\delta_{n},\;\delta_{s},\;\delta_{t}$ are the normal strain, shear strain, and tearing strain, respectively. $K_{nn}$, $K_{ss}$, and $K_{tt}$ represent the tensile stiffness, shear stiffness, and tearing stiffness, respectively, while $K_{ns}\;$ = $\;K_{nt\;}$ = $K_{st\;}$ = 0, due to the thin thickness of the cohesive element, neglecting the normal deformation [41]. The quadratic nominal stress criterion is used for damage initiation. The expression is as follows:

(2) $${\left\{\frac{\langle \sigma_{n}\rangle }{\sigma_{n}^{0}}\right\}}^{2}+{\left\{\frac{\sigma_{s}}{\sigma_{s}^{0}}\right\}}^{2}+{\left\{\frac{\sigma_{t}}{\sigma_{t}^{0}}\right\}}^{2}=1$$

In the expression, $\sigma_{n}^{0}$, $\sigma_{s}^{0}$ and $\sigma_{t}^{0}$ represent the damage initiation stresses in the three directions of the cohesive element, and $\langle \sigma_{n}\rangle$ is defined as follows:

(3) $$\langle \sigma_{n}\rangle =\left\{\begin{array}{c} \sigma_{n},\;\sigma_{n}>0\\ 0\;,\;\sigma_{n}<0 \end{array}\right.$$

when Equation (2) is satisfied, the cohesive element starts to experience damage, and then the stiffness degrades. The intrinsic relationship is as follows ($\delta_{i}^{0}<\delta_{n}<\delta_{i}^{0},i=n,s,t$):

(4) $$\left\{\begin{array}{c} \sigma_{n}\\ \sigma_{s}\\ \sigma_{t} \end{array}\right\}=\left(1-D\right)\left[\begin{array}{ccc} K_{nn} & & \\ & K_{ss} & \\ & & K_{tt} \end{array}\right]\left\{\begin{array}{c} \delta_{n}\\ \delta_{s}\\ \delta_{t} \end{array}\right\}$$

where *D* ranges from [0, 1], with 0 indicating no damage to the material and 1 indicating complete failure of the material. When the bonding interface separation exceeds the damage initiation value (when the stress reaches its peak), the bonding interface enters the softening stage. The linear softening damage variable *D* is defined as follows:

(5) $$D=\frac{\delta_{m}^{f}\left(\delta_{m}-\delta_{m}^{f}\right)}{\delta_{m}\left(\delta_{m}^{f}-\delta_{m}^{0}\right)}$$

where $\delta_{m}^{0}$ represents the effective displacement at the initiation of damage, $\delta_{m}^{f}$ represents the effective displacement at final failure, and $\delta_{m}$ represents the current effective displacement. The calculation method is as follows:

(6) $$\delta_{m}=\sqrt{{\langle \delta_{n}\rangle }^{2}+{\delta_{s}}^{2}+{\delta_{t}}^{2}}$$

Additionally, during the cohesive zone damage evolution process, it is necessary to consider the mixed-mode ratio of normal and tangential deformations. In this study, the B-K criterion based on the dissipated energy during the crack propagation process is selected [42], and its expression is as follows:

(7) $$G_{C}=G_{ΙC}+\left(G_{ΙΙC}-G_{ΙC}\right){\left(\frac{G_{S}}{G_{T}}\right)}^{\eta }$$

where η is the key index that quantifies the effect of shear mode (Type II) on the overall fracture toughness enhancement of the composite under mixed-mode (Type I + Type II) fracture [43,44]. $G_{ΙC}$ and $G_{ΙΙC}$ represent the critical fracture energy release rates for normal (Type I) and shear (Type II) modes, respectively. $G_{S}$ describes the amount of total work done by the shear traction and the corresponding relative displacement components; $G_{T}$ denotes the total work done by the normal and shear traction based on energies. $G_{C}$ is the mixed-mode critical fracture energy. As the normal and tangential displacements of the adhesive layer increase, the value of $G_{C}$ also decreases. Complete failure occurs when the cumulative dissipated energy during crack propagation reaches the total energy corresponding to the critical fracture energy.

### 3.2. CFRP Failure Criterion

Hashin’s failure criterion [45,46] incorporates in-plane damage assessment and delamination failure evaluation. This study adopts its in-plane damage criterion, with adhesive layer damage analyzed via cohesive zone modeling. Under tensile loading, laminated adhesive joints exhibit three failure modes: interlaminar peel failure, mixed-mode failure, and adhesive shear failure. To accurately simulate intralaminar matrix and fiber failure modes in laminates, this study employs the Hashin failure criterion to predict fiber failure, matrix failure, and delamination damage in CFRP laminates. The details are as follows.

Failure criterion for tensile fiber fracture ($\sigma_{11}>0)$:

(8) $${\left(\frac{\sigma_{11}}{X_{T}}\right)}^{2}+{\left(\frac{\tau_{12}}{S_{12}}\right)}^{2}+{\left(\frac{\tau_{13}}{S_{13}}\right)}^{2}=1$$

Failure criterion for compressive fiber fracture ($\sigma_{11}<0$):

(9) $${\left(\frac{\sigma_{11}}{X_{c}}\right)}^{2}=1$$

Failure criterion for tensile matrix fracture ${(\sigma }_{22}+\sigma_{33}>0)$:

(10) $${\left(\frac{\sigma_{22}+\sigma_{33}}{Y_{T}}\right)}^{2}+\frac{\left({\tau^{2}}_{23}-\sigma_{22}\sigma_{33}\right)}{{S^{2}}_{12}}+{\left(\frac{\tau_{12}}{S_{12}}\right)}^{2}+{\left(\frac{\tau_{13}}{S_{13}}\right)}^{2}=1$$

Failure criterion for compressive matrix fracture ${(\sigma }_{22}+\sigma_{33}<0)$:

(11) $$\begin{array}{c} \left(\frac{\sigma_{22}+\sigma_{33}}{Y_{T}}\right)\left[{\left(\frac{Y_{T}}{{2S}_{23}}\right)}^{2}-1\right]+{\left[\frac{\sigma_{22}+\sigma_{33}}{{2S}_{23}}\right]}^{2}+\frac{{{(\tau }^{2}}_{23}-\sigma_{22}\sigma_{33})}{{S^{2}}_{23}}+{\left(\frac{\tau_{12}}{S_{12}}\right)}^{2}+\\ {\left(\frac{\tau_{13}}{S_{13}}\right)}^{2}=1 \end{array}$$

where $\sigma_{11}$, $\sigma_{22}$, and $\sigma_{33}$ represent the normal stress components of the element in the fiberdirection, transverse direction, and thickness direction, respectively; $\tau_{12}$, $\tau_{23}$, and $\tau_{13}$ denote the three shear stress components of the element; $S_{12}$, $S_{13}$, and $S_{23}$ represent the shear strength components of CFRP in the corresponding directions; $X_{T}$, $Y_{T}$, denote the tensile strengths of the composite material in the fiber and transverse directions; and $X_{C}$, $Y_{C}$ denote the compressive strengths of CFRP in the fiber and transverse directions.

### 3.3. Mechanism of Embedded Bolt Connection

The bolt load calculation for the multi-bolt connection system is performed using an analytical method based on rigid body mechanics. It is assumed that the stiffness of the clamped components (such as connection plates) in the embedded bolt connection is much greater than that of the bolt shank [47], and their own deformation can be neglected. Therefore, under the action of bending moments, the additional axial load carried by each bolt depends solely on the position of the bolt in the connection cross-section and the overall geometric distribution of the bolt group [48]. The transverse shear force is mainly transmitted through the friction at the contact surface, while the normal separation force, perpendicular to the contact surface, is evenly distributed among all the bolts. To ensure uniform load distribution among the bolts, the load distribution formula can be used for calculation.

The second moments of area of the bolt cross-section about the *X*- and *Y*-axes, $I_{xx}$ and $I_{yy}$, as well as the product of inertia about the *X*–*Y*-axes, $I_{xy}$, can be expressed as follows:

(12) $$I_{xx}=A\sum_{i=1}^{n_{s}}y_{i}^{2}$$

(13) $$I_{yy}=A\sum_{i=1}^{n_{s}}x_{i}^{2}$$

(14) $$I_{xy}=A\sum_{i=1}^{n_{s}}x_{i}y_{i}$$

where *A* is the cross-sectional area of a single bolt, $n_{s}$ is the total number of bolts, and $x_{i}$ and $y_{i}$ are the coordinates of the *i*-th bolt in the *X* and *Y* directions of the cross-sectional coordinate system, respectively. All the above geometric parameters are calculated with respect to the defined local *X*–*Y* cross-sectional coordinate system.

The load distribution of the bolts is calculated using the following equation:

(15) $$F_{z,i}=A\left(\frac{M_{yb}I_{xx}-M_{xb}I_{xy}}{I_{xx}I_{yy}-{I^{2}}_{xy}}\right)x_{i}+A\left(\frac{M_{xb}I_{yy}-M_{yb}I_{xy}}{I_{xx}I_{yy}-{I^{2}}_{xy}}\right)y_{i}$$

After being loaded, the bolts will undergo axial strain, which for each bolt is assumed to be the following:

(16) $${∆}_{\varepsilon }=\varphi \frac{F_{z,i}}{AE}$$

where $M_{xb}$ and $M_{yb}$ are the bending moments at the bolted connection, φ is the load factor, and *E* is the elastic modulus of the bolt.

Under loading, the contact force between the bolt and the hole in the composite plate is determined by the interfacial friction. It is assumed that the friction force $F_{f}$ at the contact surface can be expressed as follows:

(17) $$F_{f}=\mu N$$

The bearing failure criterion for the hole wall can be expressed as follows:

(18) $$\sigma_{b}=\frac{P}{d.t}$$

where μ is the coefficient of friction and *N* is the normal clamping force, *P* is the load applied by the bolt, and *d* is the hole diameter.

### 3.4. Finite Element Model

Finite element simulations of the CFRP adhesive damage region and the bolt tension–compression damage region were performed using Abaqus SIMULIA, a finite element analysis software from Dassault Systèmes (Abaqus 2023). It should be noted that the dimensions used for the finite element model in this study are exactly the same as those used for the experimental samples. The simulation is divided into three main stages: bolt preload analysis, external load analysis, and damage evolution analysis. The bolt preload force was applied by means of a static analysis in Abaqus/Standard. The results of the preloading step were imported into the second analysis step as initial conditions. It is worth noting that the bolt modeling ignores the threads, uses a smooth rod bolt modeling, and defines the tangential and normal behavior of all contact pairs using both the “penalty” contact method and the “hard” contact method. The friction between the threads was simulated, which greatly simplifies the simulation steps. With this two-step analysis process (static implicit preload to explicit dynamic loading), the model is able to capture the bolt–plate contact behavior, preload effect, and the nonlinear response of the connection structure under bending loads. To better analyze the adhesive layer failure and laminate damage process, a continuous damage mechanism model was established for the adhesive layer elements based on the force-separation displacement criterion and the B-K fracture criterion. The adhesive layer in the model was defined using the adhesive interface interaction method.

**Figure 8:**
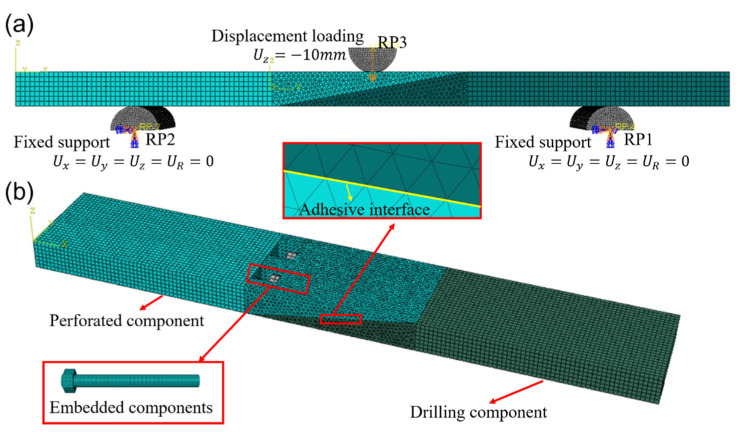
CFRP Adhesive-bolted hybrid connection finite element model: (**a**) Load and boundary conditions, (**b**) detailed meshing scheme.

The composite laminates on both sides, as well as the loading component, support parts and bolted parts are of the C3D8R hexagonal eight-node solid unit type. The bonding area of the purely glued parts adopts the C3D8R hexagonal three-dimensional eight-node solid unit type, and the bonding area and the connection area of the bolted parts adopt the C3D4 quadrilateral four-node solid unit type, and the adhesive layer is simulated by the cohesive element, and the COH3D8 eight-node three-dimensional bonding unit is used, and the adhesive layer part is built by the way of sweeping. The mesh size of the bonded area is 1 mm × 1 mm × 0.5 mm, and the mesh size of the non-bonded area is 1 mm × 1 mm × 1 mm. The total number of nodes and elements in the model is 37,501 and 87,153, respectively. For the quasi-static three-point bending experiment, regarding the boundary conditions and loading, both ends of the laminate are coupled at the midpoint of the end faces to the reference points. Boundary conditions and loads are applied to the reference points. At the reference points, the boundary conditions of the numerical model are defined as follows: the left reference point (RP1) is fully fixed, with no translation or rotation in any direction, i.e., $U_{1}=U_{2}=U_{3}=U_{R1}=U_{R2}=U_{R3}=0$. Similarly, the right reference point (RP2) is consistent with RP1. Furthermore, a compressive displacement load of 10 mm is applied at the coupling point at the center of the specimen. The finite element model and detailed meshing scheme are shown in Figure 8. This modeling approach effectively avoids local penetration phenomena and ensures the convergence of the numerical solution under complex contact conditions.

### 3.5. Mesh Convergence Analysis

In this study, a mesh sensitivity analysis was conducted for the critical damage region at the glued ply/carbon fiber composite (CFRP) joint geometry: the load–displacement variations at different mesh densities were compared using a mesh cell size of 1 mm, 0.5 mm, and 0.1 mm for two types of structures, namely, purely glued joints and glued-bolted hybrid joints, respectively. The trends of outputs such as peak load and damage displacement are observed by gradually encrypting the mesh in order to assess the dependence of the results on the mesh. The comparison results show that the simulation curves under different grid sizes basically overlap and the calculation results tend to be stable.

**Figure 9:**
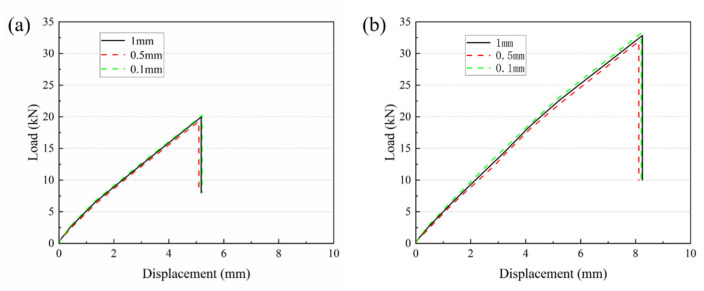
Numerical load–displacement curves for different grid sizes in the bonded area: (**a**) bonding (single-slope type), (**b**) bonding–bolted hybrid connection.

From Figure 9a,b, it can be seen that the load–displacement curves of the two types of connections almost overlap when the region is further refined to 0.5 mm and 0.1 mm, and the relative differences in the key indexes, such as peak load and damage displacement are less than 5% for each condition. This phenomenon indicates that further mesh refinement has little effect on the results [49]. Therefore, the use of a 1 mm cell size is sufficient to ensure the accuracy and stability of the simulation results.

## 4. Results and Discussion

### 4.1. Load–Displacement Relationship

#### 4.1.1. Different Adhesive Interfaces

**Figure 10:**
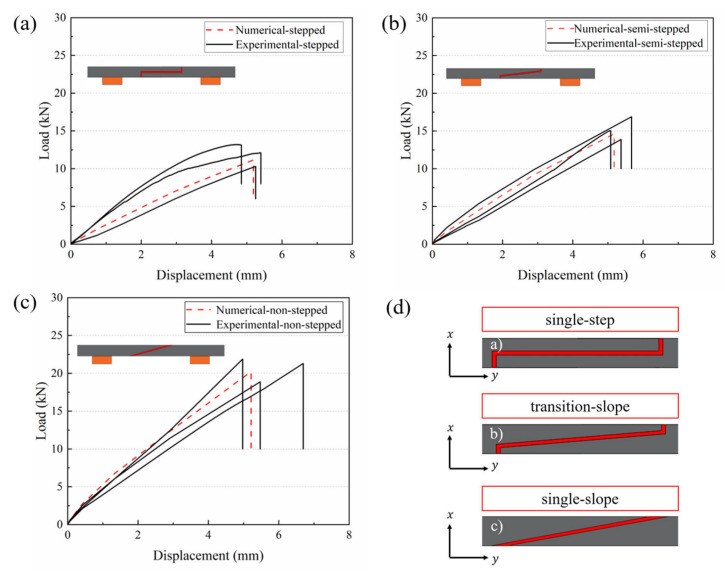
Load–displacement curves for different joint geometry structures in the bonded region: (**a**) single-step, (**b**) transition-slope, (**c**) single-slope, (**d**) bonded interface structure.

The load–displacement curves of all CFRP specimens considered in this study were obtained from quasi-static three-point bending tests. The experimental results were compared with the predictions from the finite element model, validating the effectiveness of the numerical simulation. Figure 10a–c shows the load–displacement curves of CFRP specimens with different angles of the adhesive region. The difference in displacement between the three structures is very small, and the average difference in displacement obtained experimentally for the single-slope compared to the other glued structures is in the range of 9.62% and 5.94%. It is concluded that the single-slope type has the highest bending strength and load carrying capacity under the condition of certain cutting width.

**Figure 11:**
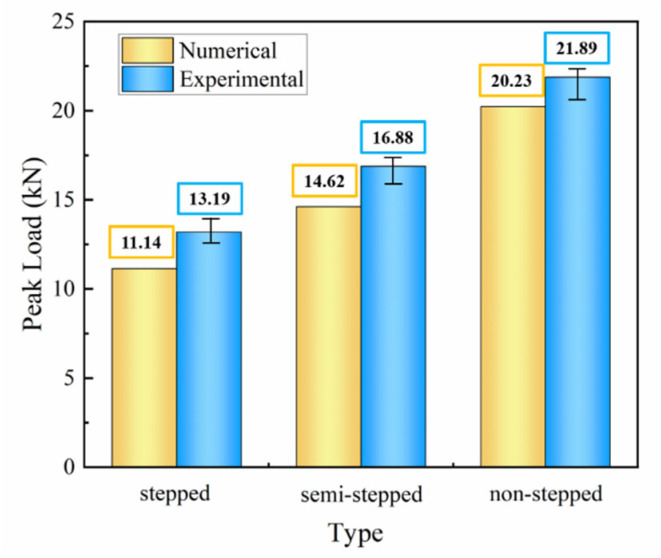
Comparison of experimental peak load and numerical peak load in CFRP bending tests with different bonding interface types.

When the experimental load reaches its peak value, the specimen fails suddenly due to its inability to withstand the applied load. Figure 11 summarizes the experimental and numerical peak loads of CFRP composite specimens with different slopes, and it is observed that the single-slope improves by 39.75% and 22.89%, respectively, as compared to other glued structures. The joint profile significantly affects the load transfer efficiency. Stepped profiles cause higher stress concentration at the edges, which in turn impacts the overall strength and failure behavior of the joint. The results show that the errors in all models are within 16%, which indicates that this finite element method can accurately and reliably predict the mechanical properties under bending loads.

#### 4.1.2. Different Connection Methods

**Figure 12:**
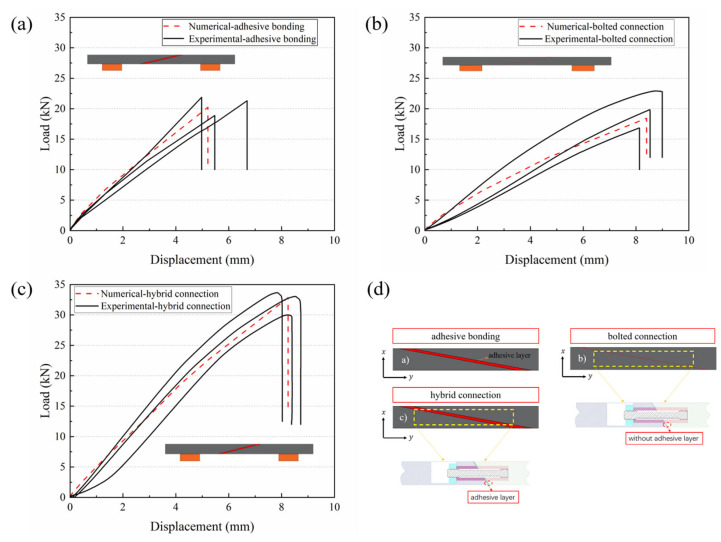
Load–displacement curves for different connection types in the bonded region: (**a**) purely glued structure, (**b**) bolted structure, (**c**) hybrid connection structure, (**d**) connection type.

Figure 12 shows the loading–displacement curves of CFRP test samples with different connection methods, and it can be seen that the advantage of the hybrid bolt-bonded connection method is more obvious, which can be manifested in the enhancement of compressive loads and bending displacements. It is worth noting that the experimental results were compared with the predicted results of the finite element model to verify the validity of the numerical simulation. Meanwhile, considering the relationship between weight and bending strength and load carrying capacity, the weight of the hybrid glued-bolted connection does not differ much compared to the other connection structures, with the weight being 0.7% higher than that of the bolted connection and 3.95% higher than that of the purely glued connection (single-slope). The highest load carrying capacity was derived for the bolted–bonded connection.

**Figure 13:**
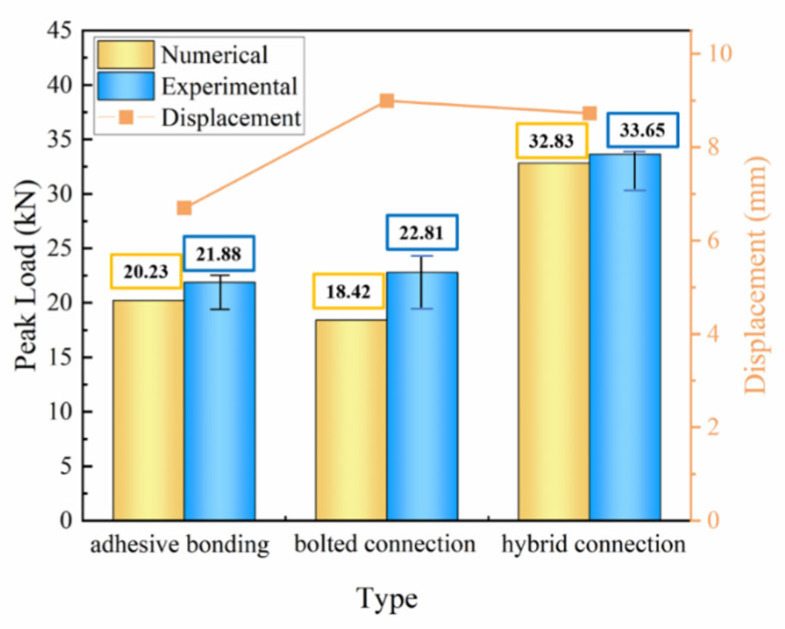
Comparison of experimental peak load and numerical peak load in CFRP bending for different connection types.

Observing Figure 13, it is evident that the peak loads are elevated by 38.38% and 43.91% compared to the other two connections, while the deflection is elevated by 31.81% compared to the glued connection, but is not much different compared to the bolted connection. This variation can be attributed to the variation in loads in different connections. It can be concluded that increasing the skew appropriately and using a hybrid bonded–bolted connection has a positive effect on improving the strength of the connection.

### 4.2. Fracture Morphology Analysis

#### 4.2.1. Macroscopic Level

**Figure 14:**
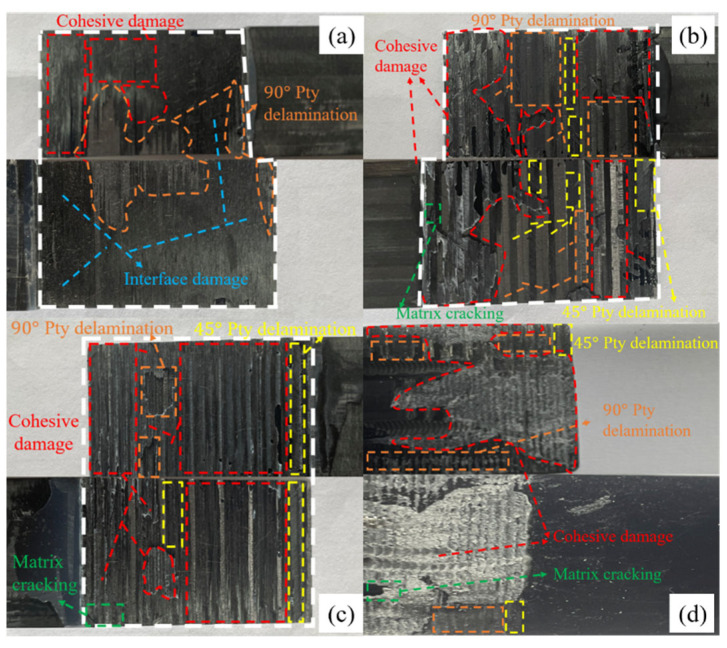
Fracture morphology under different parameter designs: (**a**) single-step, (**b**) transition-slope, (**c**) single-slope, (**d**) hybrid connection.

Under the three-point bending load, the fracture morphology of CFRP joints with different glued structural forms in this study is significantly different. As shown in Figure 14a–d, each presents a mixed failure mode but with different dominant mechanisms.

The fracture of the single-step structure (Figure 14a) is dominated by interfacial debonding (adhesive failure). The failure zone separates along almost the entire adhesive layer interface, and the fracture presents a smooth interfacial debonding surface with less adhesive residue. The interface-dominated fracture pattern suggests that the weakness of this joint is at the adhesive interface, which is consistent with its lower load-bearing strength.

The fracture of the transition-slope glued structure (Figure 14b) exhibits a mixed failure mode with the coexistence of interlayer delamination and adhesive layer cohesion damage. The fracture had adhesive residue on the surface of the matrix due to internal splitting of the adhesive layer, indicating that both the interface and the adhesive layer proper were involved in the damage. The fracture of the single-slope adhesive structure (Figure 14c) mainly manifests as cohesive failure within the composite adhesive layer, and is accompanied by cracking of the matrix resin. This phenomenon suggests that the adhesive bonding strength exceeds the strength of the composite matrix, leading to delamination failure at the adhesive interface. The fracture of the hybrid connection (Figure 14d) shows characteristics of both adhesive failure and component failure. Interlaminar delamination and cohesive failure occur simultaneously within the adhesive layer. Among them, cohesive damage dominates, with the successive failure of the adhesive layer, most of the load is transferred to the mechanical fasteners, the composite plate appears around the bolt holes with obvious matrix cracking and interlaminar delamination and other damages, and at the same time, obvious damages to the mother material of the composite material can be observed, and the cracks are further extended into the laminate along the thickness direction (90°), which leads to the delamination of the interlaminar layer and the cracking of the matrix resin. This indicates that under hybrid loading, damage propagates through the adhesive layer and affects the substrate.

#### 4.2.2. Microscopic Level

**Figure 15:**
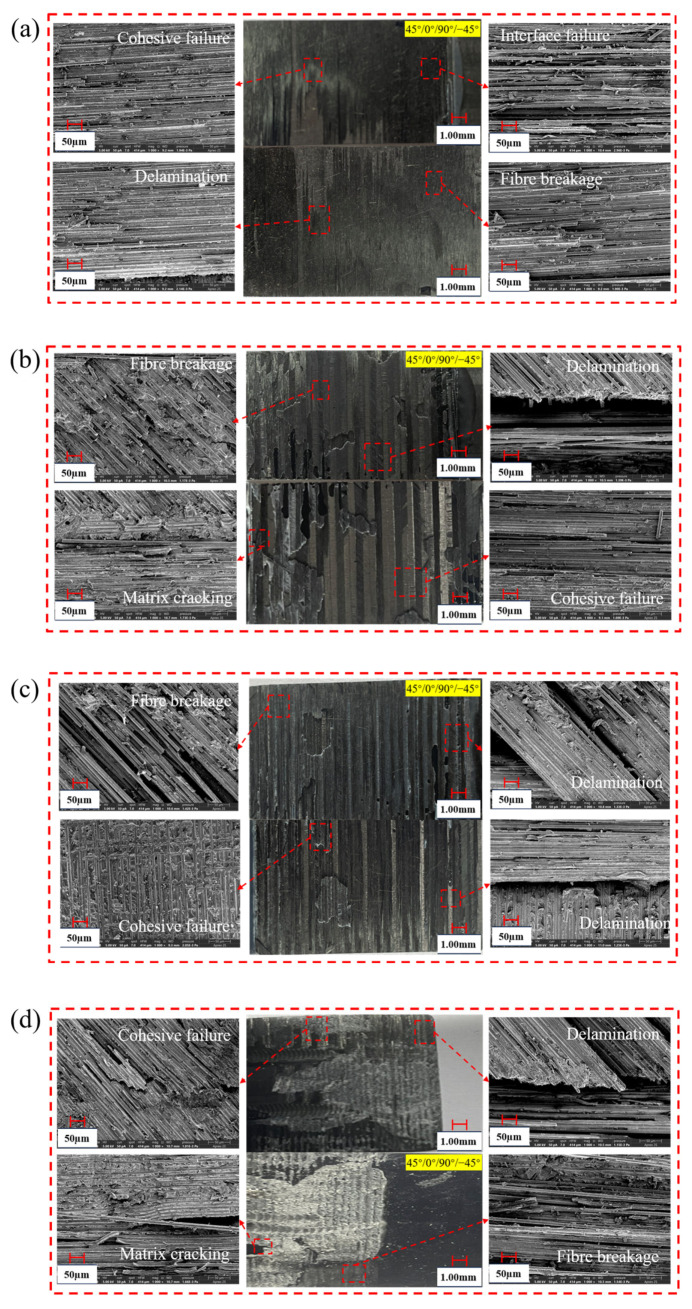
Fracture morphology of CFRP with different slopes: (**a**) single-step, (**b**) transition-slope, (**c**) single-slope, (**d**) hybrid connection.

The fracture patterns of the two different design parameters were observed using scanning electron microscopy to gain insight into their failure characteristics. It is evident from Figure 15 that cohesive damage, matrix cracking, and delamination damage are prevalent in both glued and hybrid joint structures. This observation suggests that the strength of the adhesive used exceeds the strength of the composite matrix. Fiber breaks were observed between the adhesive layers and plies, and a small amount of resin was smoothly attached to the surface of the fiber bundles. Thus, in the case of adhesive and hybrid connections used in a certain bonding region, the predominant damage pattern was delamination followed by matrix cracking, which was accompanied by limited cohesive damage within the bondline.

### 4.3. Damage Evolution

#### 4.3.1. Interfaces with Different Slopes

**Figure 16:**
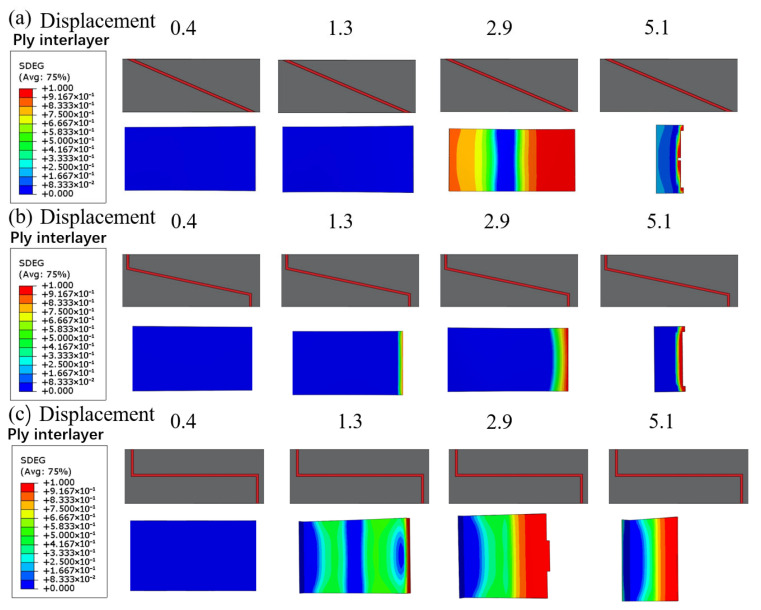
SDEG damage evolution contours in the CFRP interlaminar adhesive layer under different joint angle configurations at applied displacements of 0.4, 1.3, 2.9, and 5.1mm: (**a**) single-slope, (**b**) transition-slope bonding interface, (**c**) single-step bonding interface.

Considering the lack of effective understanding of the occurrence and development process of CFRP damage due to transient damage during the experimental process, this section explores the damage evolution process of CFRP bonded layers under different variations in the slope of the bonding interface through numerical simulations. The variable values range from 0 to 1, where 0 indicates no damage and 1 indicates complete failure. SDEG represents the damage state within the adhesive interface. Figure 16 shows the step-by-step process of damage evolution inside the adhesive layer for purely adhesive joints at three different joint angles. As seen in Figure 16, the interface geometry significantly influences the initiation and propagation of cohesive layer damage.

For the single-step bonding interface (Figure 16c), damage initiates first at the corner of the step, with a noticeable red area appearing in the adhesive layer at a relatively low displacement (around 2.9 mm), followed by rapid damage propagation along the center of the adhesive layer. The sharp geometric discontinuity at the step causes stress concentration, leading to premature failure of the adhesive layer at this location, resulting in a rapid decrease in the joint’s load-bearing capacity. Compared with the step type, damage initiation at the transition-slope bonding interface (Figure 16b) is significantly delayed under the same displacement. The adhesive layer at the transition-slope bonding interface shows only minor damage near the step transition, with the overall layer remaining intact. This indicates that the half-step design effectively mitigates the stress concentration and allows the damage to develop in a more gradual manner, resulting in a joint that exhibits a higher damage tolerance. The damage morphology at the single-slope bonding interface (Figure 16a) is intermediate between the two previously described. Since the bonding interface is a continuous slope, the adhesive layer experiences a more uniform stress distribution, though stress peaks still exist in the thin-layer region at the overlap ends. Simulation results show that damage at the single-slope interface typically begins at the thinner end and gradually propagates towards the other end of the bonding interface as displacement increases. At a displacement of approximately 5.1 mm, the entire adhesive interface experiences complete failure. The single-slope bonding interface significantly reduces the local stress peaks due to the smooth transition, delaying damage propagation, making it the highest load-bearing bonding interface design with the best overall performance among the three configurations. The single-step bonding interface, due to the most severe stress concentration, causes the adhesive interface to fail prematurely, while the damage tolerance of the transition-slope bonding interface is intermediate between the two. Experimental test results also validate the simulation conclusions.

#### 4.3.2. Hybrid Connection

**Figure 17:**
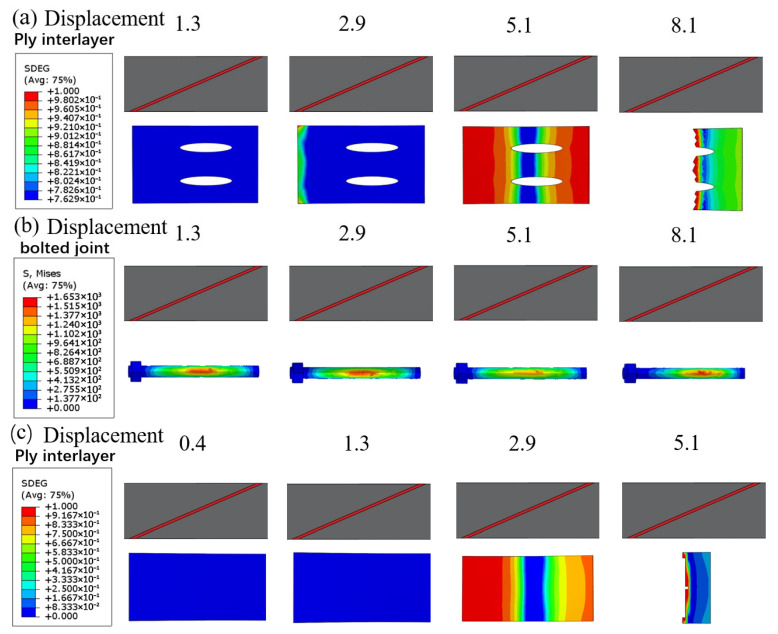
Comparative damage evolution for hybrid vs. purely bonded joints with a single-slope (single-slope) bonding interface: (**a**) SDEG contour in the adhesive layer of the hybrid joint, (**b**) von Mises stress contour in the bolt and surrounding area of the hybrid joint, and (**c**) SDEG contour in the adhesive layer of the pure adhesive joint.

Figure 17 compares the damage evolution path and failure modes of the adhesive-bolted hybrid connection and pure adhesive connection under single-slope interface conditions. It is evident that the introduction of the hybrid connection significantly alters the damage development process and the failure mechanism of the adhesive region. For the hybrid connection, cohesive damage in the adhesive layer begins to appear at an early stage.

As shown in Figure 17a, at a displacement of 1.3 mm, a localized red area appears near the bolt hole in the adhesive layer, indicating that the adhesive starts to peel off near the bolt. However, despite the early onset of damage in the adhesive layer, the mechanical constraint provided by the bolt delays the overall failure of the structure. As the load increases, the damage area of the adhesive layer in the hybrid joint gradually expands, and by approximately 5.1 mm of displacement, most of the adhesive layer has failed completely (SDEG close to 1). At this point, the bolt takes on the primary load, and the joint remains intact. Figure 17b shows that during the gradual debonding of the adhesive layer, the equivalent stress in the bolt and the surrounding composite plate significantly increases, especially around the bolt root and the hole, where higher stress concentrations appear. When the displacement reaches approximately 8.1 mm, the bolt or the surrounding structure reaches its strength limit, and at this point, the hybrid joint experiences final failure. Overall, the hybrid connection joint exhibits a two-stage failure mode: first, the gradual debonding failure of the adhesive layer, followed by the bolt bearing the load until its final fracture. This mechanism improves the final failure displacement and ultimate strength of the joint.

**Figure 18:**
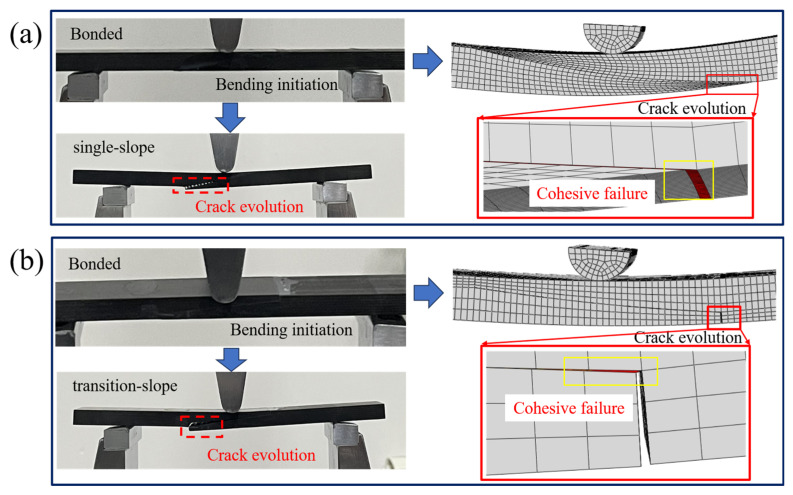

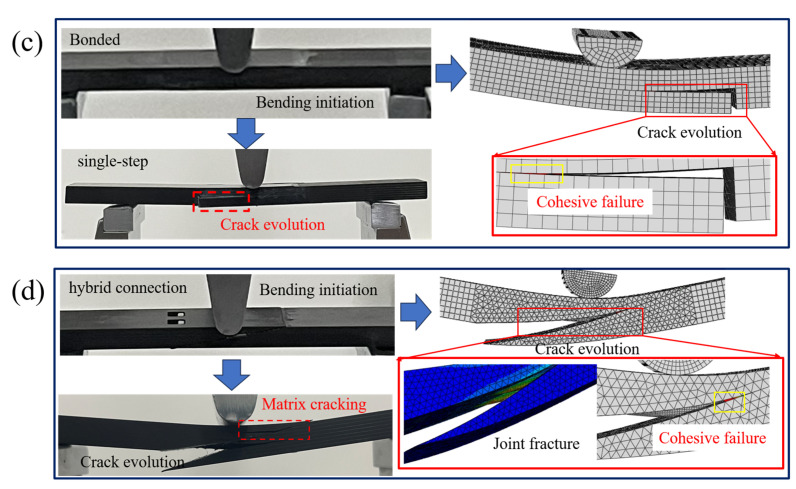
Shows various steps and trends in static flexural testing of bonded and hybrid connections: (**a**) single-slope, (**b**) transition-slope, (**c**) single-step, (**d**) hybrid connection.

As shown in Figure 18, both the experimental and simulation steps exhibit consistent trends, particularly in terms of crack evolution and fracture modes. It is evident from the figure that different bonding configurations display varying crack propagation behaviors. The corresponding simulation results accurately reflect these trends, with failure mechanisms including cohesive failure and matrix cracking observed in all test cases. Both the experimental and simulation results reveal similar fracture surfaces and crack evolution patterns, further validating the reliability of the computational model in capturing the material behavior under bending stress.

## 5. Summary and Conclusions

To study the comprehensive performance of the joint geometry, the mechanical properties of CFRP joints with different bonding interfaces and connection methods under bending loads were investigated through experimental and numerical approaches. The failure process and failure modes of the connection region were studied. Additionally, the load-bearing capacity, fracture morphology, and damage evolution of the CFRP joint structures were revealed. Based on the above work, the main conclusions are as follows:

(1) Different adhesive interfaces

The peak load of the adhesive region in pure adhesive bonding increases with the slope under a constant width. Specifically, the single-slope design exhibits the highest peak load, with peak loads obtained from numerical simulation and experiments being 20.23 kN and 21.89 kN, respectively. Compared to other adhesive structures, this represents an increase of 39.75% and 22.89%. The displacement differences between the three structures are very small, with the average experimental difference being 9.62% and 5.94%.

The study found that the lap slope has a significant impact on damage evolution. The step-type structures are dominated by interfacial debonding with no significant visual damage within the CFRP layer. The other structures are coupled failures with multiple damage types. The use of a gentler lap slope leads to a more uniform stress transition along the adhesive layer and the laminate, which delays the failure of the adhesive layer and induces more damage inside the composite.

(2) Different connection methods

The experimental and numerical simulation results for the adhesive-bolted hybrid connection specimens both show the highest peak load, with values of 32.83 kN and 33.65 kN, representing increases of 38.38% and 43.91%, respectively. The maximum difference in the average peak load obtained experimentally was 34.98% and the minimum difference was 32.21%. Compared to the single-slope pure adhesive bonding structure, the deflection increased by 31.81%.

The hybrid connection shows a more significant improvement in load-bearing capacity compared to pure adhesive bonding. It can further optimize the structural performance and damage tolerance. The fracture surface exhibits characteristics of both adhesive failure and component failure. The hybrid connection demonstrates a two-stage failure mode, while the pure adhesive bonding shows a single-stage failure mode.

(3) A predictive modeling technique has been developed to help understand the effects of different design parameters on the mechanical properties and failure mechanisms. The experimental results are in good agreement with the numerical results, validating the effectiveness of the modeling approach.

Future research can further optimize CFRP joint designs by including additional adhesive properties, joint dimensions, and bolt configurations. Additionally, it can focus on fatigue testing and full-scale validation to ensure long-term durability and reliability in real-world applications.

## Figures and Tables

**Table 3:** Mechanical properties of materials used for bolting.

Components	E/GPa	ν	$R_{t}$/MPa	$R_{m}$/MPa	Strength Grade
Bolt/Nut	210	0.31	1040	940	4.8
Bushing	210	0.31	900	750	-

## Data Availability

The raw data supporting the conclusions of this article will be made available by the authors on request.
